# Changing therapy from Glivec^® ^to a "copy" imatinib results in a worsening of chronic myeloid leukemia disease status: two case reports

**DOI:** 10.1186/1757-1626-2-9342

**Published:** 2009-12-17

**Authors:** Inas A Asfour, Shereen A Elshazly

**Affiliations:** 1 Department of Internal Medicine, Clinical Hematology Unit, Faculty of Medicine, Ain Shams University, Cairo, Egypt

## Abstract

**Introduction:**

Imatinib mesylate (Glivec^®^/Gleevec^®^) is the standard first-line therapy for the treatment of chronic myeloid leukemia due to its high hematologic, cytogenetic, and molecular response rates and favorable long-term safety profile. A copy version of imatinib is currently available in several countries. We report on two cases of CML who were originally treated with Glivec in Egypt and subsequently switched to the copy drug

**Case presentation:**

Case one was a 35-year old female with chronic myeloid leukemia in blast crisis who began treatment with combination chemotherapy and Glivec. The patient achieved and maintained a complete hematologic response and continued on Glivec 400 mg/day. In March 2007, she was switched to the copy drug In September 2007, the patient presented in hematologic relapse. At this time, treatment with chemotherapy in combination with Glivec 400 mg/day was resumed. The patient quickly achieved, and maintained, complete hematologic response on Glivec 400 mg/day. The second patient was a 64-year old male with chronic myeloid leukemia in blast crisis who began treatment with truncated chemotherapy in combination with Glivec 400 mg/day. After 6 months, the patient achieved a partial hematologic response and continued on alternating cycles of chemotherapy with continuous administration of Glivec 400 mg/day. The patient received Glivec from January 2006 to February 2007, after which time he was switched to the copy drug. In November 2007, he presented with upper gastrointestinal bleeding and multiple gastric erosions and died the same day.

**Conclusion:**

The safety and efficacy of the copy drug has not been established in randomized clinical trials. It is unknown whether patients, who respond to Glivec and then switch to copy versions of imatinib, will tolerate the copy drug and maintain their response.

## Introduction

Chronic myeloid leukemia (CML) is a clonal myeloproliferative disease characterized by the presence of the Philadelphia chromosome. The Philadelphia chromosome is formed from the rearrangement of the long arms of chromosomes 9 and 22, resulting in the constitutively active protein tyrosine kinase, BCR-ABL [1,2]. Without treatment, CML progresses within several years from a chronic phase (CML-CP) to an accelerated phase, and ultimately to a blast crisis (CML-BC) which may be myeloid or lymphoid in origin and rapidly leads to death without intensive treatment [2].

The introduction of imatinib mesylate (Glivec/Gleevec; Novartis Pharmaceuticals), a tyrosine kinase inhibitor of BCR-ABL, has revolutionized the treatment of CML. Imatinib is widely accepted as the standard of care for the first-line treatment of CML due to its well-documented clinical activity resulting in durable responses and prolonged survival [3-6]. Seven year follow-up of the phase III licensing trial, the International Randomized Study of Interferon and STI571 (IRIS) showed sustained responses, high survival rates, and favorable long-term safety for patients randomized to first-line imatinib, with a cumulative complete cytogenetic response (CCyR) rate of 82%, rate of freedom from progression to AP/BC of 93%, 81% event-free survival (EFS) and 86% overall (OS) survival rate for this group [7].

Unfortunately, cost and access to medication can be a barrier to patient compliance. Recently, a copy-version of imatinib, has become available in several countries. Unlike a generic version of a pharmaceutical that must demonstrate bioequivalence to the branded drug by a regulatory agency, this copy-drug claims to be "comparable" to imatinib but has not been rigorously tested to determine its purity and efficacy. As a result of lower pricing and easy access, often not requiring a prescription, some patients and healthcare agencies have substituted this copy-version for imatinib in some countries.

Here, we report 2 cases of patients diagnosed with CML-CP, both treated in Egypt, at Ain Shams University Hospital's clinical hemato-oncology unit, who were originally treated with branded Glivec and subsequently switched to a copy version of imatinib.

## Case presentation

### Case report 1

The first patient was a 35-year old Egyptian female (Arabic), diagnosed with CML-CP in 2004. She was initially treated with hydroxyurea, resulting in control of her disease for approximately 2 years. She presented to our clinic in September 2006 with complaints of increasing fatigue and bruising. On clinical exam she exhibited several skin bruises and subcutaneous bleeds, huge splenomegaly 4 cm below the costal margin hepatomegaly, and no palpable lymphadenopathy. Her initial laboratory assessment revealed a total leukocyte count of 12.7 × 10^9^/L, with 32% blast cells in the peripheral blood smear, hemoglobin (Hgb) concentration of 7.3 g/dL, and platelet count of 66 × 10^9^/L. Bone marrow aspiration revealed a hypercellular marrow due to infiltration, with 84% heterogeneous blast cells exhibiting diffuse negativity for myeloperoxidase stain and suppression of all other marrow elements.

On immunophenotypic examination the isolated blast cells were more than 90% positive for CD34, HLA-DR, and CD10. Eighty-four percent of isolated blast cells were CD19 positive, with 44% positive for CD13 and 54% positive for CD33. Cytogenetic examination using the G-band method displayed a Ph^+ ^clone, with subclonal evolution acquiring rearranged chromosomes 4 and 13. Fluorescent in situ hybridization (FISH) showed a Philadelphia chromosome in all metaphase cells and 80% in interphase cells. Other laboratory results were within normal limits, including a negative viral screen.

A diagnosis of CML-BC was made and the patient began treatment with combination chemotherapy (consisting of prednisone, asparaginase, vincristine, doxorubicin, cyclophosphamide, cytarabine, mercaptopurine, and methotrexate) and Glivec [8]. The patient initially experienced a hematologic response (HR) with subsequent examination after induction therapy showing a complete hematologic response (CHR), defined as < 2% blasts on bone marrow exam. The patient was maintained on Glivec 400 mg daily and remained in CHR. Cytogenetic and molecular analyses were not performed on this patient. In March 2007 the patients was switched to a copy-version of imatinib, at the same dose of 400 mg daily due to unavailability of Glivec at the patient's hospital. Three months after the change in medication, an increase in liver transaminases was noted on a routine follow-up visit, necessitating a dose reduction.

In September 2007, the patient presented in hematologic and central nervous system (CNS) relapse, with a total leukocyte count of 7.8 × 10^9^/L, with a differential of 77% neutrophils, 2% myelocytes, 2% metamyelocytes, 15% lymphocytes, and 2% immature cells. Bone marrow aspiration revealed 20% blast cells which were myeloperoxidase negative. Immunophenotypic examination demonstrated similar markers to those observed at initial diagnosis. At this time, treatment utilizing the initial strategy of systemic chemotherapy in combination with branded Glivec 400 mg daily was resumed. The patient also received intrathecal chemotherapy for treatment of the CNS relapse.

**Figure 1:**
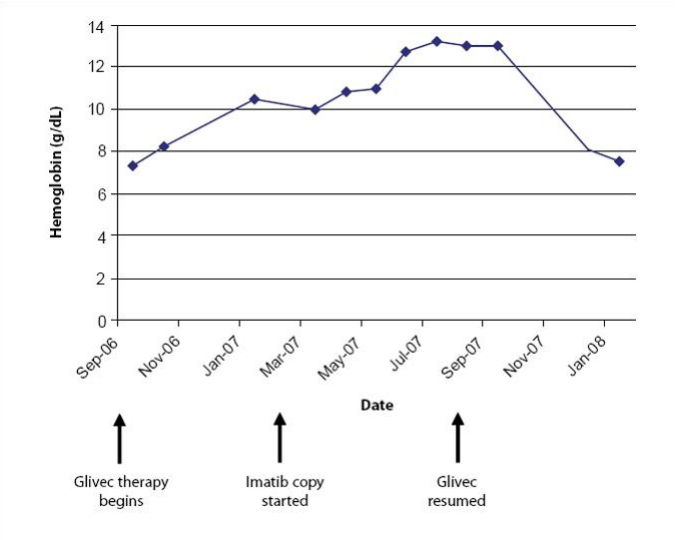
**Case 1: Changes in hemoglobin**.

**Figure 2:**
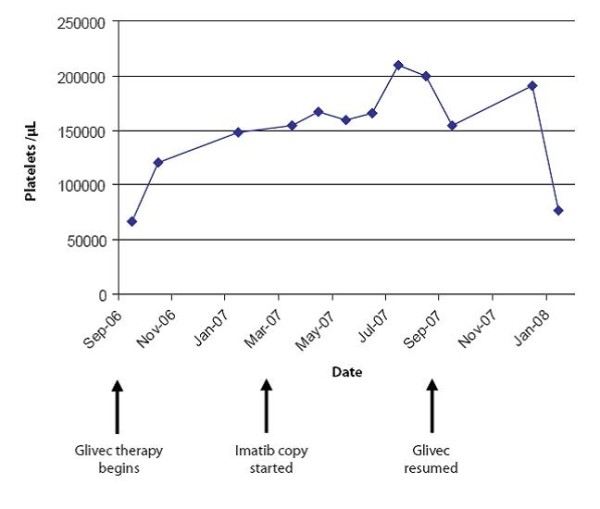
**Case 1: Changes in platelets**.

**Figure 3:**
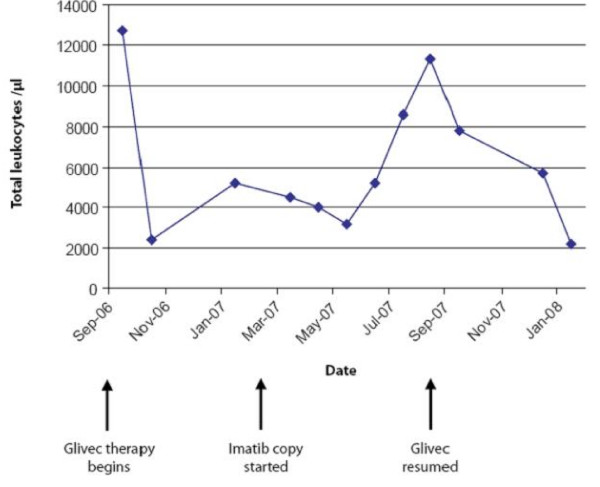
**Case 1: Changes in total leukocyte count**.

On day 28 after reinduction with chemotherapy and Glivec, a CHR was documented. The patient declined stem cell transplantation during her first remission and again at this time. As of April 2008, seven months after reinduction with chemotherapy and Glivec, the patient remained in CHR on 400 mg daily Glivec. Figure 1, Figure 2 and Figure 3 illustrate the changes in her hematologic indices over the course of treatment.

### Case report 2

The second patient was a 64-year-old Egyptian male (Arabic), diagnosed with CML in 1998. The patient initially received treatment with hydroxyurea and subcutaneous interferon. He presented to our clinic in December, 2005 with complaints of increasing fatigue, abdominal girth, and bruising. Physical examination revealed splenomegaly reaching the umbilicus and a moderate hepatomegaly of 4 cm/below the costal margin. Initial laboratory results revealed a total leukocyte count of 200 × 10^9^/L^3 ^with 66% blast cells in the peripheral blood smear, Hgb concentration of 8 g/dL, and platelet count of 622 × 10^9^/mL^3^. Bone marrow aspiration revealed a hypercellular marrow due to infiltration with 73% blast cells, with an increase in eosinophils and basophils, constituting 14% of marrow cells.

On immunophenotypic examination, the isolated blast cells showed an acute myeloid pattern with a major monocytic component. Cytogenetic examination using the G-band method revealed all cells were Philadelphia chromosome positive. Other laboratory results showed positivity for hepatitis C antibodies, but the patient exhibited normal liver and renal function.

A diagnosis of CML-BC was made and the patient began treatment with subcutaneous cytarabine and oral etoposide in combination with branded Glivec 400 mg daily. The patient was unable to receive conventional therapy due to poor performance status. After six months, the patient achieved a partial hematologic response with bone marrow examination showing a hypercellular marrow with 10% blast cells. The patient continued on alternating cycles of oral etoposide and subcutaneous cytarabine with continuous administration of Glivec 400 mg daily. The patient received branded Glivec from January 2006 to February 2007, after which he was switched to a copy-version of imatinib 400 mg daily due to unavailability of Glivec at the patient's hospital. In November 2007, the patient presented with upper gastrointestinal bleeding, and endoscopy revealed multiple small gastric erosions. His hematological profile showed a total leukocyte count of 188 × 10^9^/L with 36% blast cells and marked absolute neutrophilia and monocytosis in the peripheral blood smear, Hgb concentration of 5.8 g/dL, and platelet count of 539 × 10^9^/L. The patient was given blood transfusions and admitted to the hospital for further evaluation. The next day his total leukocyte count doubled to 400 × 10^9^/L, and his coagulation profile showed markers consistent with disseminated intravascular coagulation with hyperkalemia. Unfortunately, the patient expired the same day.

## Discussion

The advent of Glivec has greatly improved the treatment of CML. Both patients presented here achieved adequate responses while undergoing treatment with Glivec. Subsequently, both patients switched to a copy version of imatinib. Although other explanations might be offered (eg, lack of compliance, advanced disease), the loss of hematologic response in both patients and CNS relapse and gastric erosions in case 1 and 2, respectively, were temporally related to the change from branded Glivec to the copy.

Patient safety with copy drugs needs to be considered before making a switch from Glivec. Typical clinical results are well known with branded Glivec, the only drug that has been tested in clinical trials and has shown cumulative CCyR and EFS rates of 82% and 81%, respectively, after 7 years of follow-up [7]. No data exists to show that copies are safe and effective. Physicians need to be aware of the risks associated with copy drugs, and make these risks known to their patients. These data underscore the importance of maintaining proper medication with careful monitoring in CML patients to achieve, maintain, and sustain hematologic responses. For this reason, Novartis established the Glivec International Patient Assistance Program (GIPAP). This program is one of the most generous patient assistance programs in existence and is currently available in more than 80 countries worldwide.

## Conclusions

Imatinib mesylate is the current standard first-line therapy for CML. A copy version of the drug is available in some countries, for which patient data are limited, and this drug has not been shown to be safe and effective in randomized clinical trials. Major guidelines recommend indefinite continuation of imatinib treatment unless loss of response or intolerance is experienced [5,6].

## Consent

Written informed consent was obtained from the patients or their guardians for publication of this case series and any accompanying images. A copy of the written consent is available for review by the Editor-in-Chief of this journal.

## Competing interests

The authors declare that they have no competing interests.

## Authors' contributions

IA postulated the concept and designed the study; SA interpreted the data, reviewed the literature, and prepared the manuscript. All authors have read and approved the final manuscript.
